# Direct oral anti-Xa anticoagulants versus warfarin in newly diagnosed atrial fibrillation and CKD: the Korean National Health Insurance Data

**DOI:** 10.3389/fmed.2023.1212816

**Published:** 2023-09-15

**Authors:** Youn Kyung Kee, Hee Jung Jeon, Jieun Oh, Tae-Hyun Yoo, Dongwoo Kang, Jungkuk Lee, Dong Ho Shin

**Affiliations:** ^1^Department of Internal Medicine, College of Medicine, Kangdong Sacred Heart Hospital, Hallym University, Seoul, Republic of Korea; ^2^Department of Internal Medicine, College of Medicine, Yonsei University, Seoul, Republic of Korea; ^3^Data Science Team, Hanmi Pharm. Co., Ltd., Seoul, Republic of Korea

**Keywords:** atrial fibrillation, chronic kidney disease, direct oral anti-Xa anticoagulants, effectiveness, safety, warfarin

## Abstract

**Introduction:**

Despite the benefits of direct oral anti-Xa anticoagulants (DOACs), the risk–benefit profile of DOAC therapy compared to warfarin therapy in patients with non-valvular atrial fibrillation (AF) and chronic kidney disease (CKD), including end-stage renal disease (ESRD), is uncertain.

**Methods:**

We conducted a retrospective study using the Korea National Health Insurance Database from 2013 to 2018. We evaluated patients with incident non-valvular AF and CKD. The primary and secondary effectiveness outcomes were ischemic stroke and all-cause mortality. The primary safety outcomes included intracranial hemorrhage, gastrointestinal bleeding, and extracranial or unclassified major bleeding.

**Results:**

Among the 1,885 patients evaluated, 970 (51.5%) initiated warfarin therapy, and 915 (48.5%) initiated DOAC therapy. During a mean follow-up period of 23.8 months, there were 293 and 214 cases of ischemic stroke and all-cause death, respectively. Kaplan–Meier survival analysis showed significantly lower all-cause mortality in DOAC users than in warfarin users. In multivariate Cox regression analyses, DOAC therapy had a hazard ratio for all-cause mortality of 0.41 (95% CI, 0.30–0.56; *p* < 0.001) compared to warfarin therapy. Additionally, DOAC therapy significantly reduced intracranial hemorrhage and gastrointestinal bleeding.

**Discussion:**

Our study demonstrates that DOAC therapy has a better risk–benefit profile than warfarin therapy in patients with AF and CKD. Further well-designed clinical trials are needed to clarify the benefits of DOACs in this patient population.

## Introduction

Atrial fibrillation (AF) is the most common sustained cardiac arrhythmia and is associated with an increased risk of ischemic stroke and mortality compared to patients maintaining a sinus rhythm (1–3). AF occurs more often in patients with advanced age, hypertension, diabetes, heart failure, and vascular disease (4, 5). Chronic kidney disease (CKD) frequently occurs in aging patients with comorbidities such as diabetes or hypertension (6, 7). AF and CKD share several common risk factors and have a direct relationship. The prevalence of AF usually increases as kidney function deteriorates. In patients with pre-dialysis CKD who develop end-stage renal disease (ESRD), the prevalence of AF can reach approximately 15%. This is over three times the prevalence in age-matched controls (8). In addition, ischemic stroke clearly increases mortality in patients with CKD compared to those without CKD (9).

The prevention of ischemic stroke is the cornerstone of AF management. Therefore, clinical guidelines recommend oral anticoagulant therapy in patients with AF (3, 10). However, patients with CKD are not only at a higher risk of ischemic stroke but also at an increased risk of bleeding even without oral anticoagulants (11). Despite differing recommendations in AF guidelines (12–14), warfarin therapy is still widely used in patients with AF and CKD. Warfarin is eliminated by hepatic metabolism, suggesting that drug dose adjustment may not be necessary for patients with CKD (12, 15). Nevertheless, considering warfarin’s narrow therapeutic window and its susceptibility to multiple drug and food interactions, interpretation of dose adjustment solely based on estimated glomerular filtration rate (eGFR) may be an over-simplified approach under uremia and downregulation of CYP450 in these patients (11, 16). Several clinical studies have shown that patients with CKD require lower warfarin dosages to maintain therapeutic anticoagulation compared to patients with normal kidney function (17, 18). Another caveat to warfarin therapy is that a higher risk of major bleeding events for greater fluctuations in international normalized ratio (INR) values, or any given INR value, was commonly observed in these patients (19, 20). In addition, there are concerns about increased vascular calcification and calciphylaxis with warfarin, as it reduces the function of vitamin K-dependent vascular calcification inhibitors, such as matrix G1a proteins (21, 22). Observational studies have not clearly demonstrated the benefits of VKAs when compared to not undergoing any treatment in patients with non-valvular AF undergoing hemodialysis (23–25).

Over a decade ago, direct oral anti-Xa anticoagulants (DOACs) emerged as promising alternatives to vitamin K antagonists (VKAs), demonstrating a similar efficacy but lower bleeding risk (26–28). This led to expectations of a superior risk–benefit profile for DOACs, particularly in patients with atrial fibrillation (AF) and chronic kidney disease (CKD). Landmark trials such as RE-LY, ROCKET-AF, and ARISTOTLE have established the effectiveness of DOACs in the general population with AF (26–28). However, these trials had limited representation of CKD patients, especially those with advanced disease, resulting in ongoing uncertainty regarding the ideal anticoagulation strategy in this high-risk group. Several observational studies have examined the use of DOACs in patients with CKD, as summarized in a recent meta-analysis (29). This study has provided valuable insights into the safety and effectiveness of DOACs in this population. However, there are still important gaps in our understanding. To further contribute to this body of research, our study uses the Korean National Health Insurance Database to focus on a specific subgroup of CKD patients, aiming to explore long-term outcomes associated with DOAC use.

## Materials and methods

### Data sources

This retrospective cohort study was performed using data from the Republic of Korea National Health Insurance Service (NHIS). The NHIS is a nationwide universal healthcare insurer that covers the entire Korean population. All clinics, hospitals, and pharmacies in Korea are required to participate in the NHIS, and they are reimbursed for their services through the NHIS after filing claims electronically. Claims are accompanied by data regarding demographic characteristics, diagnostic codes classified by the 10th revision of the International Classification of Diseases (ICD-10), procedure codes, and prescription records. The NHIS constructs a nationwide claims database called the National Health Insurance Database (NHID), which contains data on death compiled by Statistics Korea. These data are publicly available at https://nhiss.nhis.or.kr/bd/ab/bdaba000eng.do. We constructed comprehensive datasets of baseline demographics, clinical covariates on diagnosis, study medications, and outcomes based on these data.

### Study population

Among patients who had chronic kidney disease (CKD) aged 18 years or older from January 1, 2013, to December 31, 2017, we considered patients newly diagnosed with AF as eligible. We included all patients diagnosed with AF, regardless of whether they were diagnosed in an inpatient or outpatient setting. The target study cohort included patients with non-valvular AF who had initiated oral anticoagulant therapy, either DOACs or warfarin, since 2014. We began our sampling from 2013 to identify baseline comorbidities and looked for treatment in 2014. Exclusion criteria included the use of anticoagulants for less than 90 days, cancer patients, or previous history of pulmonary thromboembolism, deep vein thrombosis, ischemic stroke, or hemorrhage (intracranial hemorrhage, gastrointestinal bleeding, extracranial or unclassified major bleeding). It is important to note that most doctors are unwilling to remove an existing diagnostic code in the Korean healthcare system because they may not be reimbursed for their claims if they do not enter an appropriate diagnostic code. As a result, once a diagnostic code is entered, it is almost always maintained. Therefore, to extract diagnostic codes as outcomes, the patient had to be established as event-naive. Supplementary Table 1 shows ICD-10 codes for diagnosing diseases to extract the study population. The diagnostic codes used to identify atrial fibrillation, stroke, and hemorrhage have been validated and used in a previous study (30).

### Data collection and study design

Baseline characteristics and clinical outcomes of the patients were evaluated according to DOAC users or warfarin users. The index date was defined as the first date of prescription of DOACs or warfarin. Baseline demographic characteristics were obtained from insurance claims at the index date, and baseline comorbidities referred to any claim with diagnostic codes at the index date or in the prior year at the index date. Supplementary Table 2 shows the ICD-10 codes for comorbidities. Patients with all stages of CKD were stratified into pre-dialysis CKD and ESRD groups. Pre-dialysis CKD was identified by insurance claims with diagnostic codes, and ESRD was identified by insurance claims with the diagnostic code and regular dialysis (hemodialysis and/or peritoneal dialysis) procedure codes (Supplementary Table 2). The Charlson comorbidity index (CCI), CHA_2_DS_2_-VASc score, and modified HAS-BLED (mHAS-BLED) score were calculated to evaluate the general comorbidity status, ischemic stroke risk, and major bleeding risk, respectively. Because factors such as H (uncontrolled hypertension with systolic blood pressure > 160 mmHg), L (labile international normalized ratios), and D (alcohol) were not known on the index date, they were not included in the calculation of the mHAS-BLED score. Therefore, the maximum mHAS-BLED score was 5 (23, 31, 32). The use of medications, defined as lasting for >90 cumulative days after the index date, was identified by insurance claims with prescription records. We also collected data on the use of calcium channel blockers (CCB) and beta blockers (BB), as these medications are commonly used in patients with AF and could potentially influence outcomes. The follow-up period was from the index date until the first occurrence of any clinical outcomes, the date of ceasing or changing anticoagulant from warfarin to DOACs, or the end date of the study period (December 31, 2018), based on whichever came first. This variability in follow-up duration is inherent in real-world observational studies and reflects the reality of clinical practice. Despite this variability, the statistical methods used for data analysis, such as time-to-event analyses, are designed to handle this inconsistency and provide valid results. Notably, if patients with pre-dialysis CKD had changed to patients with ESRD during the study follow-up period, these patients were defined as patients with pre-dialysis CKD and were followed up at the time of change. In other words, they were censored when dialysis was begun.

### Outcome assessment

We assessed the following outcomes: the primary effectiveness outcome was an ischemic stroke, including transient ischemic attack, and the secondary effectiveness outcome was all-cause mortality. The primary safety outcomes included intracranial hemorrhage, gastrointestinal bleeding, and extracranial or unclassified major bleeding. Using diagnostic codes, we defined major bleeding according to the criteria established by the International Society on Thrombosis and Haemostasis (ISTH) (33). Furthermore, we considered a bleeding event to be ‘major’ if it necessitated hospitalization. Outcomes were also recognized from insurance claims using the diagnostic codes (Supplementary Table 1).

### Statistical analysis

Categorical variables are presented as frequencies with percentages and continuous variables as means and standard deviations (SDs). Differences between treatment groups were evaluated by the *t*-test for continuous variables and by the *χ*^2^ or Fisher exact test for categorical variables. The cumulative incidence of outcomes between DOAC therapy and warfarin therapy was calculated using the Kaplan–Meier estimation method with the log-rank test. Event-free survival time and time to events during the total observation period were compared between the two groups using Cox proportional hazards models. We performed a series of models that sequentially adjusted for categories of potential explanatory variables: Model 1 (age, sex, dialysis dependency, CCI), Model 2 (Model 1 + baseline use of medications [antiplatelets, beta blocker, calcium channel blocker, renin-angiotensin-aldosterone system blocker, diuretics, statin]), and Model 3 (Model 2 + CHA_2_DS_2_-VASc score, mHAS-BLED score). Additionally, effectiveness and safety outcomes between the two groups were assessed in important clinical subgroups: age < 65 vs. age ≥ 65, male vs. female, pre-dialysis CKD vs. ESRD, CHA_2_DS_2_-VASc ≤3 vs. CHA_2_DS_2_-VASc >3 (higher ischemic stroke risk), and mHAS-BLED ≤2 vs. mHAS-BLED >2 (higher major bleeding risk). Lastly, as a supplementary analysis to enhance the robustness of our findings, we employed Propensity Score Matching (PSM). The propensity score was determined using a multivariable logistic regression model with baseline covariates (Table 1). Patients with the nearest propensity scores between Warfarin users and DOAC users were matched using a 1:1 scheme without replacement using a greedy algorithm. All statistical tests were evaluated using a two-tailed 95% confidence interval (CI), and statistical significance was set at *p* < 0.05. All analyses were performed using SAS software (version 9.3, SAS Institute) and R programming language version 3.3.1.

**Table 1:** Baseline characteristics of subjects.

	Total (*n* = 1,885)	Warfarin user (*n* = 970)	DOAC user (*n* = 915)	*p*-value
Age, y	71.1 ± 11.1	68.4 ± 12.0	73.7 ± 10.1	<0.001
Male, n (%)	1,060 (56.2)	573 (59.1)	487 (53.2)	<0.001
Comorbid disease, n (%)				
Pre-dialysis CKD	1,510 (80.1)	655 (67.5)	855 (93.4)	<0.001
ESRD	375 (19.9)	315 (32.5)	60 (6.6)	<0.001
Diabetes mellitus	1,340 (71.1)	675 (69.6)	665 (72.7)	0.14
Congestive heart failure	1,073 (56.9)	551 (56.8)	522 (57.0)	0.93
Myocardial infarction	222 (11.8)	111 (11.4)	111 (12.1)	0.67
Peripheral vascular disease	773 (41.0)	361 (37.2)	412 (45.0)	0.001
Charlson’s comorbidity index	4.39 ± 2.03	4.08 ± 2.02	4.69 ± 2.04	<0.001
CHA_2_DS_2_-VASc	4.91 ± 1.66	4.64 ± 1.63	5.17 ± 1.68	<0.001
CHA_2_DS_2_-VASc ≤3, n (%)	428 (22.7)	257 (26.5)	171 (18.7)	
CHA_2_DS_2_-VASc >3, n (%)	1,457 (77.3)	713 (73.5)	744 (81.3)	
mHAS-BLED^*^	2.90 ± 0.85	2.72 ± 0.90	3.07 ± 0.79	<0.001
mHAS-BLED^*^ ≤2, n (%)	148 (7.9)	107 (11.0)	41 (4)	
mHAS-BLED^*^ >2, n (%)	1,737 (92.1)	863 (89.0)	874 (96)	
Medication, n (%)				
Aspirin	902 (47.9)	449 (46.3)	453 (49.5)	0.17
Other antiplatelets	367 (19.5)	178 (18.4)	189 (20.7)	0.22
BB	937 (49.7)	521 (53.7)	416 (45.5)	<0.001
CCB	1,163 (61.7)	593 (61.1)	570 (62.3)	0.64
RASB	1,342 (71.2)	687 (70.8)	655 (71.6)	0.72
Diuretics	1,056 (56.0)	536 (55.3)	520 (56.8)	0.52
Statin	968 (51.4)	465 (47.9)	503 (55.0)	0.002

DOACs, Direct Oral Anti-Xa Anticoagulants; RASB, renin-angiotensin-aldosterone system blocker; CCB, calcium channel blocker; BB, beta blocker. ^*^Modified HAS-BLED.

## Results

### Study population

**Figure 1:**
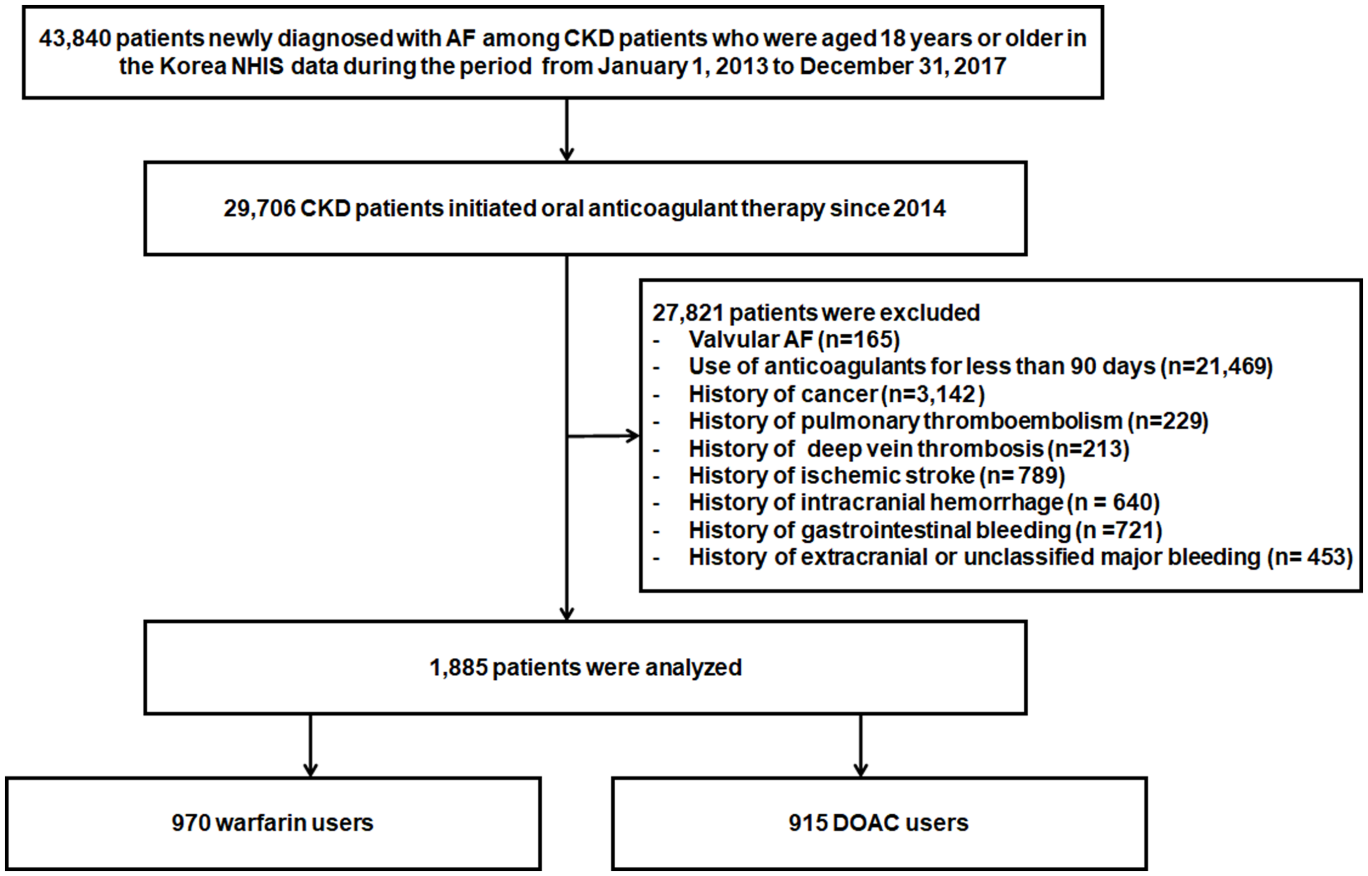
Flowchart of study population enrollment. AF, atrial fibrillation; DOACs, Direct Oral Anti-Xa Anticoagulants; NHIS, National Health Insurance Service.

Among patients with chronic kidney disease (CKD) aged 18 years or older from January 1, 2013, to December 31, 2017, 43,840 patients newly diagnosed with AF were identified in the Korean NHIS database. A total of 29,706 patients initiated anticoagulant therapy, DOACs, or warfarin and were eligible for inclusion since 2014. Of these patients, 27,821 patients were excluded for the following reasons: valvular AF, such as those with rheumatic mitral stenosis or surgical heart valves (*n* = 165); the use of anticoagulants for less than 90 days (*n* = 21,469); cancer patients (*n* = 3,142); previous history of pulmonary thromboembolism (*n* = 229), deep vein thrombosis (*n* = 213), ischemic stroke (*n* = 789), intracranial hemorrhage (*n* = 640), gastrointestinal bleeding (*n* = 721), and extracranial or unclassified major bleeding (*n* = 453). Thus, a total of 1,885 patients were included in the analysis (Figure 1).

### Direct oral anti-Xa anticoagulants

Prescribed drug doses according to the type of DOAC, as shown in Supplementary Table 3, were as follows: low-dose apixaban (2.5 mg twice daily), low-dose rivaroxaban (15 mg once daily), and low-dose edoxaban (30 mg twice daily) were prescribed in 60, 183, and 174 patients, respectively.

### Baseline characteristics

The baseline characteristics are shown in Table 1. The mean age was 71.1 ± 11.1 years, and 1,060 (56.2%) were men. Among the 1,885 patients, 970 patients (51.5%) initiated warfarin therapy, whereas 915 (48.5%) initiated DOAC therapy. Compared to warfarin users, DOAC users were significantly older and had a lower proportion of men. Although DOAC users had a lower proportion of patients with ESRD than warfarin users (6.6% vs. 32.5%, *p* < 0.001), DOAC users had higher CCI, CHA_2_DS_2_-VAS_C_ scores_,_ and mHAS-BLED score than warfarin users. Meanwhile, although the use of beta blockers was more frequent in DOAC users than in warfarin users, the use of aspirin, other antiplatelets, renin-angiotensin-aldosterone system blockers, calcium channel blockers, and diuretics was comparable between the two groups.

### Ischemic stroke

**Figure 2:**
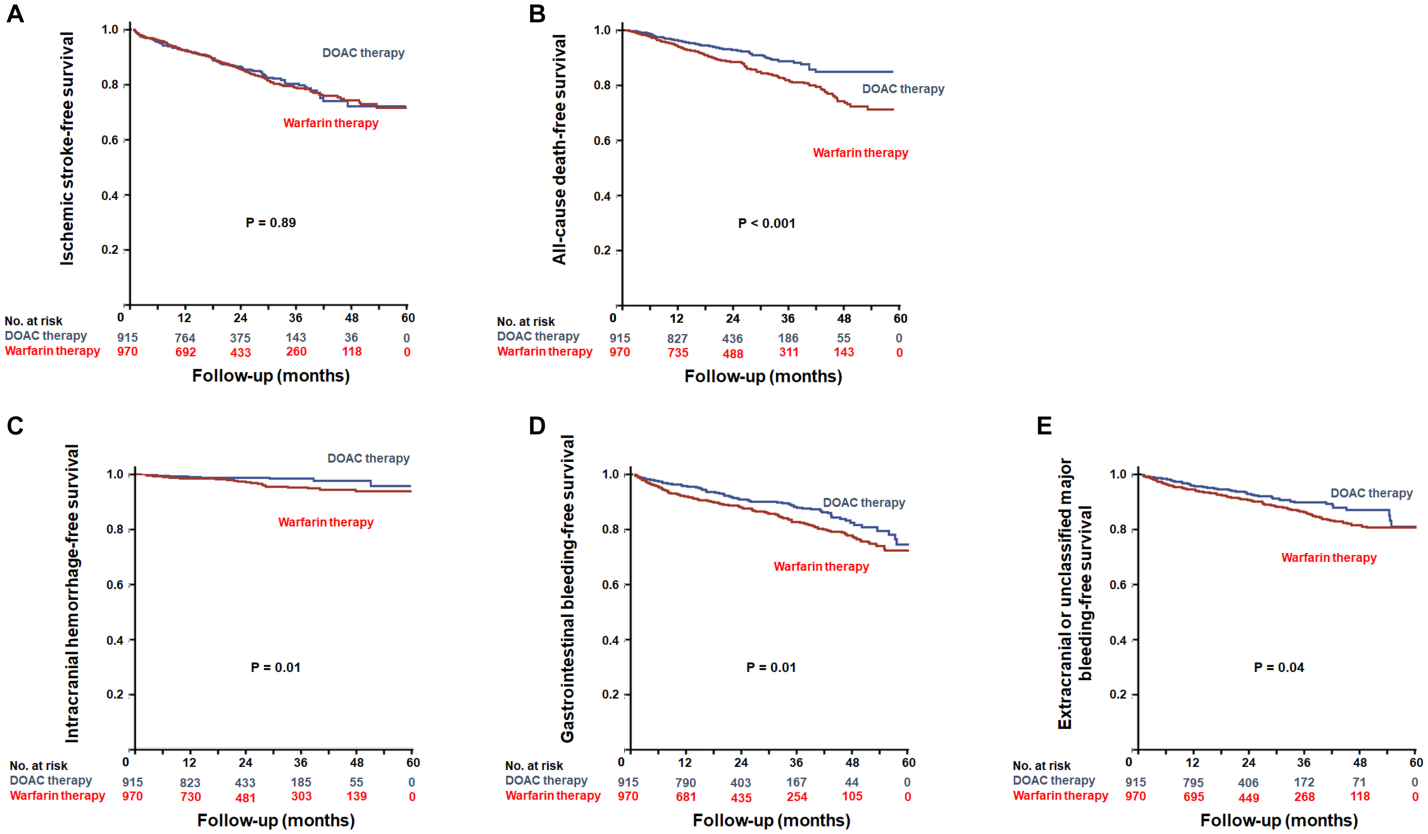
Kaplan–Meier plots for efficacy outcomes between warfarin users and DOAC users. Event-free survival of ischemic stroke **(A)** and all-cause death **(B)**. Kaplan–Meier plots for safety outcomes between warfarin users and DOAC users. Event-free survival of intracranial hemorrhage **(C)**, gastrointestinal bleeding **(D)**, and extracranial or unclassified major bleeding **(E)**. DOACs, Direct Oral Anti-Xa Anticoagulants.

**Figure 3:**
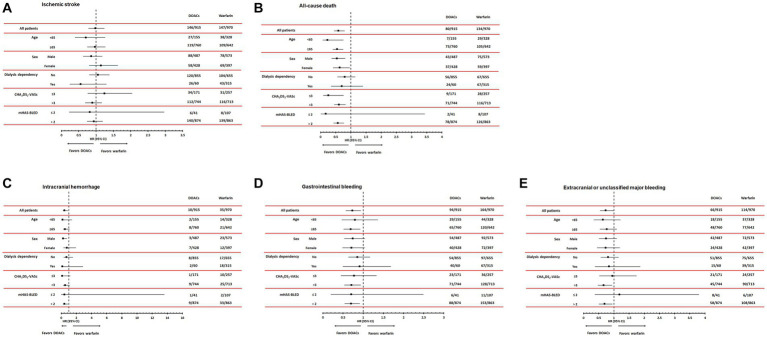
Subgroup analyses according to age, sex, dialysis dependency, CHA_2_DS_2_-VASc, and modified HAS-BLED score comparing hazard ratios of efficacy outcomes, including ischemic stroke **(A)** and all-cause mortality **(B)** and safety outcomes, including intracranial hemorrhage **(C)**, gastrointestinal bleeding **(D)**, and extracranial or unclassified major bleeding **(E)**. DOACs, Direct Oral Anti-Xa Anticoagulants.

During a mean follow-up duration of 23.8 ± 15.2 months, ischemic stroke occurred in 293 patients (15.5%). The incidence of ischemic stroke was comparable between warfarin and DOAC users (1.73 vs. 1.96 per 1,000 patient-years, *p* = 0.89). In Kaplan–Meier survival analysis and Cox regression analyses, there was also no difference in ischemic stroke between warfarin and DOAC users (Figure 2A and Table 2). Of note, when the entire study population was divided into important clinical subgroups, in patients with ESRD and pre-dialysis CKD, there was no difference in ischemic stroke between warfarin and DOAC users (Figure 3A).

**Table 2:** Primary and secondary efficacy outcomes.

Outcome	Group (n)	Events (n)	Person-year (per 1,000)	Unadjusted HR. (95% CI)	*p* value	Adjusted^*^ HR. (95% CI)	*p* value	Adjusted^**^ HR. (95% CI)	*p* value	Adjusted^***^ HR. (95% CI)	*p* value
Ischemic stroke	Warfarin user										
	970	156	1.96	ref		ref		ref		ref	
	DOAC user										
	915	137	1.73	0.98 (0.78–1.24)	0.89	0.83 (0.65–1.05)	0.12	0.82 (0.64–1.05)	0.12	0.82 (0.64–1.05)	0.12
All-cause death	Warfarin user										
	970	141	2.12	ref		ref		ref		ref	
	DOAC user										
	915	73	1.93	0.59 (0.45–0.70)	<0.001	0.42 (0.31–0.56)	<0.001	0.12 (0.31–0.57)	<0.001	0.41 (0.30–0.56)	<0.001

^*^Model 1: adjusted for age, sex, dialysis dependency, and CCI; ^**^Model 2: adjusted for model 1 + baseline use of medications; ^***^Model 3: adjusted for model 3 + CHA_2_DS_2_-VASc score + modified HAS-BLED score. HR, hazard ratio; DOACs, Direct Oral Anti-Xa Anticoagulants; CI, confidence interval.

### All-cause death

A total of 214 all-cause deaths occurred during the follow-up period. The incidence of all-cause death occurred less frequently in DOAC users than in warfarin users (1.93 vs. 2.15 per 1,000 patient-years, *p* < 0.001). Kaplan–Meier survival analysis showed that all-cause mortality was significantly lower in DOAC users than in warfarin users (Figure 2B). In Cox regression analyses, DOAC therapy significantly reduced all-cause mortality compared with warfarin therapy. The impact of DOAC therapy on all-cause death remained significant even after adjustments for age, sex, dialysis dependency, medications, CCI, CHA_2_DS_2_-VAS_C_ score_,_ and mHAS-BLED score (hazard ratio [HR], 0.41; 95% CI, 0.30 to 0.56; *p* < 0.001) (Table 2). However, the subgroup analysis showed that DOAC therapy did not reduce all-cause mortality compared to warfarin therapy in patients with pre-dialysis CKD and ESRD, respectively (Figure 3B).

### Hemorrhage

During the follow-up period, intracranial hemorrhage, gastrointestinal bleeding, and extracranial or unclassified major bleeding occurred in 45 (2.4%), 258 (13.7%), and 180 (9.5%) patients, respectively. The incidences of intracranial hemorrhage (1.92 vs. 2.12 per 1,000 patient-years, *p* = 0.02), gastrointestinal bleeding (1.82 vs. 1.93 per 1,000 patient-years, *p* = 0.02), and extracranial or unclassified major bleeding (1.84 vs. 1.99 per 1,000 patient-years, *p* = 0.04) were significantly lower in DOAC users than warfarin users. Kaplan–Meier survival analysis also showed that the occurrence rate of intracranial hemorrhage, gastrointestinal bleeding, and extracranial or unclassified major bleeding was significantly lower in DOAC users than in warfarin users (Figures 2C–E). Meanwhile, compared with warfarin therapy, DOAC therapy significantly reduced only intracranial hemorrhage and gastrointestinal bleeding in univariate and multivariate Cox regression analyses (Table 3). Especially in the subgroup analysis, DOAC therapy significantly reduced hemorrhagic risk compared to warfarin therapy in patients with higher major bleeding risk (mHAS-BLED>2). However, DOAC therapy did not reduce hemorrhagic risk compared to warfarin therapy in patients with pre-dialysis CKD and ESRD (Figures 3C–E).

**Table 3:** Primary safety outcomes.

Outcome	Group (n)	Events (n)	Person-year (per 1,000)	Unadjusted HR. (95% CI)	*p* value	Adjusted^*^ HR. (95% CI)	*p* value	Adjusted^**^ HR. (95% CI)	*p* value	Adjusted^***^ HR. (95% CI)	*p* value
Intracranial hemorrhage	Warfarin user										
	915	32	2.12	ref		ref		ref		ref	
	DOAC user										
	970	13	1.92	0.45 (0.23–0.85)	0.02	0.48 (0.24–0.94)	0.03	0.49 (0.25–0.97)	0.04	0.48 (0.24–0.96)	0.04
Gastrointestinal bleeding	Warfarin user										
	915	154	1.93	ref		ref		ref		ref	
	DOAC user										
	970	104	1.82	0.73 (0.57–0.94)	0.02	0.64 (0.49–0.84)	0.001	0.64 (0.49–0.84)	0.001	0.65 (0.50–0.85)	0.002
Extracranial or unclassified Major bleeding	Warfarin user										
	915	107	1.99	ref		ref		ref		ref	
	DOAC user										
	970	73	1.84	0.73 (0.57–0.94)	0.04	0.75 (0.54–1.02)	0.07	0.75 (0.56–1.04)	0.08	0.78 (0.56–1.07)	0.12

^*^Model 1: adjusted for age, sex, dialysis dependency, and CCI;^**^Model 2: adjusted for model 1 + baseline use of medications;^***^Model 3: adjusted for model 3 + CHA_2_DS_2_-VASc score + modified HAS-BLED score. HR, hazard ratio; DOACs, Direct Oral Anti-Xa Anticoagulants; CI, confidence interval; CCI, Charlson comorbidity index.

### Outcomes after propensity score matching

To address potential confounding, we further analyzed the primary and secondary efficacy and safety outcomes after propensity score matching. Post-propensity score matching, both groups had 120 patients each (Supplementary Table 4). For the outcome of ischemic stroke, DOAC users had a comparable risk to warfarin users (HR: 0.94, 95% CI: 0.50–1.75, *p* = 0.82). However, the risk of all-cause death was significantly lower in DOAC users compared to warfarin users (HR: 0.53, 95% CI: 0.30–0.95, *p* = 0.03) (Supplementary Table 5). The safety profile post-matching revealed that DOAC users had a significantly reduced risk of intracranial hemorrhage (HR: 0.40, 95% CI: 0.16–0.99, *p* = 0.05), gastrointestinal bleeding (HR: 0.50, 95% CI: 0.29–0.86, *p* = 0.001), and extracranial or unclassified major bleeding (HR: 0.53, 95% CI: 0.30–0.93, *p* = 0.03) when compared to warfarin users (Supplementary Table 6).

## Discussion

Using a large-scale, nationwide population-based database, this study evaluated ischemic stroke, all-cause mortality, and the incidence of hemorrhage in AF patients with CKD according to DOAC or warfarin therapy. In particular, subgroup analyses of AF patients with ESRD were conducted because of the concern that DOAC therapy or warfarin therapy might not convey the same safety and effectiveness in patients with ESRD compared with pre-dialysis CKD.

The present study showed that DOAC therapy reduced hemorrhagic risk and all-cause mortality in patients with AF and CKD compared to warfarin therapy. However, there was no significant difference between DOAC therapy and warfarin therapy in reducing the risk of ischemic stroke in these patients. Meanwhile, in subgroup analyses of AF patients with ESRD, there were no significant differences between DOAC therapy and warfarin therapy concerning ischemic stroke, all-cause mortality, and hemorrhagic risk.

These findings align with those of several similar observational studies. For instance, Chen et al. found that DOACs were associated with a lower risk of stroke or systemic embolism and intracranial hemorrhage than warfarin in patients with CKD and AF (34). This is in line with our results showing a reduced hemorrhagic risk and all-cause mortality with DOAC therapy compared to warfarin in AF and CKD patients. A study focusing on apixaban use in end-stage kidney disease patients with AF found that apixaban was associated with lower rates of major bleeding than warfarin, which is consistent with our findings in the subgroup analyses of AF patients with ESRD (35). Finally, a study by Makani et al. concluded that in patients with concomitant renal impairment and AF, DOAC use was safe and effective with a lower risk of mortality across all stages of renal impairment (36). This supports our findings that DOAC therapy had a better risk–benefit profile than warfarin therapy in patients with AF and CKD. While our findings are consistent with these studies, there may be discrepancies with other studies in the field. These discrepancies could be due to differences in study design, patient populations, or other factors, and further research is needed to reconcile these differences.

Over a decade ago, DOACs were recommended as alternatives in the guidelines. Compared with VKAs, DOACs have a more predictable anticoagulant response, a more rapid onset/offset of action, and fewer drug or food interactions (37). In addition, although the dose of VKAs to maintain therapeutic anticoagulation is determined on an individual basis, DOACs are administered at a fixed dose and do not require regular coagulation monitoring (37). Because of this difference between VKAs and DOACs, DOACs are associated with lower bleeding risk than VKAs (26–28). Many clinical studies have shown that DOAC therapy had a better risk–benefit profile in patients with non-valvular AF (37–40). Despite these advantages of DOACs over VKAs, DOAC therapy requires caution in patients with CKD. Though the extent varies, all DOACs rely on the kidney for elimination, necessitating dose adjustment in CKD patients (11, 41). Unfortunately, there is no standardized test for monitoring DOACs to confirm the maintenance of therapeutic anticoagulation in these patients after dose adjustment.

Despite certain limitations of DOACs in patients with CKD, post-analyses of key effectiveness randomized control trials (RCTs) in AF demonstrated equivalent or superior effectiveness and safety of DOACs compared with VKAs, provided that the recommended dose reductions are made (42–44). In line with these findings, in the present study, we observed superior safety of DOAC therapy compared to warfarin therapy, even though DOAC users had a higher bleeding risk than warfarin users. On the other hand, evidence of the safety and effectiveness of DOACs in patients with ESRD is lacking because the key RCTs excluded these patients (26–28, 45). For this reason, clinicians are reluctant to use DOACs, and warfarin is commonly prescribed despite the high bleeding risk. Of note, in this study, warfarin users were also more common than DOAC users in patients with ESRD. Clinicians generally prescribe subtherapeutic doses of warfarin, as its bleeding risk is notably higher in ESRD patients than in those with CKD. In fact, Laura et al. reported that these patients were three times more likely to be below the target INR range (46). This finding suggests that clinicians are conservative with their warfarin dosing strategies in patients with ESRD; therefore, these patients more frequently experience suboptimal effectiveness with warfarin compared to DOACs. Recently, a valkyrie trial clearly showed that in patients with ESRD, a reduction in rivaroxaban significantly decreased cardiovascular disease and major bleeding compared to VKA (47). However, our subgroup analyses indicated no reduced risk of all-cause mortality and hemorrhage with DOAC therapy compared to warfarin among ESRD patients. This outcome may be attributed to the relatively limited sample size of ESRD patients in our study.

Our study has several strengths. One of the key strengths is our ‘as treated’ analysis approach. In our study, we censored patients if anticoagulants were stopped. This approach provides a more accurate representation of the real-world use of these medications, as it takes into account changes in treatment over time. This is particularly important in a long-term observational study like ours, where treatment strategies may change due to a variety of factors, including changes in patients’ health status, side effects, or new clinical guidelines.

Our study faced several limitations. The first was that the study relied on the insurance claims data, meaning all variables and outcomes were sourced from an electronic database without the benefit of a detailed review of clinical presentations. Vital information such as CKD stage, serum creatinine, and estimated glomerular filtration rate (eGFR) were unobtainable. This prevented us from accurately categorizing CKD based on direct eGFR measures and assessing the appropriateness of DOAC dose reduction based on eGFR. Secondly, the lack of laboratory data and information on alcohol consumption might have introduced bias in calculating mHAS-BLED scores and determining the adequacy of warfarin dosage. Lastly, the study encountered difficulties in data extraction, specifically regarding other major bleeding sites as defined by the ISTH criteria (33). Structural limitations of the insurance database prevented us from capturing the full scope of the ISTH’s definition of major bleeding. These challenges inherent in the insurance database’s structure and data extraction capabilities are inherent limitations to our research methodology.

Despite these limitations, a relatively large number of incident patients were enrolled in the current study and had a long follow-up period. Based on these major strengths, the findings showed that DOAC therapy had a better risk–benefit profile than warfarin therapy in patients with AF and CKD. Further well-designed clinical trials are needed to clarify the benefits of DOACs in patients with AF and CKD.

In conclusion, our study provides valuable insights into the safety and effectiveness of DOACs in patients with AF and CKD. Our findings suggest that DOACs may offer a potential safety advantage over warfarin, even in patients with a higher baseline risk. These findings have important implications for clinical practice and highlight the need for further research in this area.

## Data availability statement

The data analyzed in this study is subject to the following licenses/restrictions: the permission to access the data is restricted to research and subjects to consent of national health insurance. Requests to access these datasets should be directed to http://nhiss.nhis.or.kr.

## Author contributions

YK and DS were involved in the conception and design of the study. DK and JL performed the data analysis. All authors were involved in interpretation of data analysis and manuscript preparation.

## Funding

This research was generously supported by a grant from the Kangdong Sacred Heart Hospital Fund under grant number CDM2022-03. We express our sincere gratitude for their financial assistance, which made this study possible.

## Conflict of interest

DK and JL are employed by Hanmi Pharm. Co., Ltd.

The remaining authors declare that the research was conducted in the absence of any commercial or financial relationships that could be construed as a potential conflict of interest.

## Publisher’s note

All claims expressed in this article are solely those of the authors and do not necessarily represent those of their affiliated organizations, or those of the publisher, the editors and the reviewers. Any product that may be evaluated in this article, or claim that may be made by its manufacturer, is not guaranteed or endorsed by the publisher.

## References

[1] Hylek EM Go AS Chang Y Jensvold NG Henault LE Selby JV . Effect of intensity of oral anticoagulation on stroke severity and mortality in atrial fibrillation. N Engl J Med. (2003) 349:1019–26. doi: 10.1056/NEJMoa022913 12968085

[2] Camm AJ Kirchhof P Lip GY Schotten U Savelieva I Ernst S . Guidelines for the management of atrial fibrillation: the task force for the management of atrial fibrillation of the european society of cardiology (Esc). Eur Heart J. (2010) 31:2369–429. doi: 10.1093/eurheartj/ehq278 20802247

[3] January CT Wann LS Calkins H Chen LY Cigarroa JE Cleveland JC Jr . 2019 Aha/Acc/Hrs focused update of the 2014 Aha/Acc/Hrs guideline for the management of patients with atrial fibrillation: a report of the American College of Cardiology/American Heart Association task force on clinical practice guidelines and the heart rhythm society. J Am Coll Cardiol. (2019) 74:104–32. doi: 10.1016/j.jacc.2019.01.011 30703431

[4] Schnabel RB Sullivan LM Levy D Pencina MJ Massaro JM D'Agostino RB Sr . Development of a risk score for atrial fibrillation (framingham heart study): a community-based cohort study. Lancet. (2009) 373:739–45. doi: 10.1016/s0140-6736(09)60443-8 19249635 PMC2764235

[5] Soliman EZ Prineas RJ Go AS Xie D Lash JP Rahman M . Chronic kidney disease and prevalent atrial fibrillation: the chronic renal insufficiency cohort (Cric). Am Heart J. (2010) 159:1102–7. doi: 10.1016/j.ahj.2010.03.027 20569726 PMC2891979

[6] James MT Hemmelgarn BR Tonelli M. Early recognition and prevention of chronic kidney disease. Lancet. (2010) 375:1296–309. doi: 10.1016/s0140-6736(09)62004-3 20382326

[7] Coresh J Selvin E Stevens LA Manzi J Kusek JW Eggers P . Prevalence of chronic kidney disease in the United States. JAMA. (2007) 298:2038–47. doi: 10.1001/jama.298.17.2038 17986697

[8] Olesen JB Lip GY Kamper AL Hommel K Køber L Lane DA . Stroke and bleeding in atrial fibrillation with chronic kidney disease. N Engl J Med. (2012) 367:625–35. doi: 10.1056/NEJMoa1105594 22894575

[9] Kelly DM Ademi Z Doehner W Lip GYH Mark P Toyoda K . Chronic kidney disease and cerebrovascular disease: consensus and guidance from a Kdigo controversies conference. Stroke. (2021) 52:e328–46. doi: 10.1161/strokeaha.120.029680 34078109

[10] Lip GYH Banerjee A Boriani G Chiang CE Fargo R Freedman B . Antithrombotic therapy for atrial fibrillation: chest guideline and expert panel report. Chest. (2018) 154:1121–201. doi: 10.1016/j.chest.2018.07.040 30144419

[11] Law JP Pickup L Townend JN Ferro CJ. Anticoagulant strategies for the patient with chronic kidney disease. Clin Med. (2020) 20:151–5. doi: 10.7861/clinmed.2019-0445 PMC7081809 32188649

[12] January CT Wann LS Alpert JS Calkins H Cigarroa JE Cleveland JC Jr . 2014 Aha/Acc/Hrs guideline for the management of patients with atrial fibrillation: a report of the American College of Cardiology/American Heart Association task force on practice guidelines and the heart rhythm society. J Am Coll Cardiol. (2014) 64:e1–e76. doi: 10.1016/j.jacc.2014.03.022 24685669

[13] Kirchhof P Benussi S Kotecha D Ahlsson A Atar D Casadei B . 2016 Esc guidelines for the management of atrial fibrillation developed in collaboration with eacts. Eur Heart J. (2016) 37:2893–962. doi: 10.1093/eurheartj/ehw210 27567408

[14] Verma A Cairns JA Mitchell LB Macle L Stiell IG Gladstone D . 2014 focused update of the Canadian cardiovascular society guidelines for the management of atrial fibrillation. Can J Cardiol. (2014) 30:1114–30. doi: 10.1016/j.cjca.2014.08.001 25262857

[15] Ansell J Hirsh J Hylek E Jacobson A Crowther M Palareti G. Pharmacology and management of the vitamin K antagonists: american college of chest physicians evidence-based clinical practice guidelines (8th edition). Chest. (2008) 133:160S–98S. doi: 10.1378/chest.08-0670, PMID: 18574265

[16] Jain N Reilly RF. Clinical pharmacology of Oral anticoagulants in patients with kidney disease. Clin J Am Soc Nephrol. (2019) 14:278–87. doi: 10.2215/cjn.02170218 29802125 PMC6390909

[17] Sakaan SA Hudson JQ Oliphant CS Tolley EA Cummings C Alabdan NA . Evaluation of warfarin dose requirements in patients with chronic kidney disease and end-stage renal disease. Pharmacotherapy. (2014) 34:695–702. doi: 10.1002/phar.1445 24851819

[18] Harder S . Renal profiles of anticoagulants. J Clin Pharmacol. (2012) 52:964–75. doi: 10.1177/0091270011409231 21610202

[19] Limdi NA Beasley TM Baird MF Goldstein JA McGwin G Arnett DK . Kidney function influences warfarin responsiveness and hemorrhagic complications. J Am Soc Nephrol. (2009) 20:912–21. doi: 10.1681/asn.2008070802 19225037 PMC2663833

[20] Limdi NA Limdi MA Cavallari L Anderson AM Crowley MR Baird MF . Warfarin dosing in patients with impaired kidney function. Am J Kidney Dis. (2010) 56:823–31. doi: 10.1053/j.ajkd.2010.05.023 20709439 PMC2963672

[21] Yalamanchili V Reilly RF. Does the risk exceed the benefit for anticoagulation in end-stage renal disease patients with nonrheumatic atrial fibrillation? Semin Dial. (2011) 24:387–8. doi: 10.1111/j.1525-139X.2011.00885.x 21851395

[22] Eiser AR . Warfarin, calciphylaxis, atrial fibrillation, and patients on dialysis: outlier subsets and practice guidelines. Am J Med. (2014) 127:253–4. doi: 10.1016/j.amjmed.2013.08.033 24333618

[23] Yoon CY Noh J Jhee JH Chang TI Kang EW Kee YK . Warfarin use in patients with atrial fibrillation undergoing hemodialysis: a Nationwide population-based study. Stroke. (2017) 48:2472–9. doi: 10.1161/strokeaha.117.017114 28801476

[24] Randhawa MS Vishwanath R Rai MP Wang L Randhawa AK Abela G . Association between use of warfarin for atrial fibrillation and outcomes among patients with end-stage renal disease: a systematic review and meta-analysis. JAMA Netw Open. (2020) 3:e202175. doi: 10.1001/jamanetworkopen.2020.2175 32250434 PMC7136833

[25] Van Der Meersch H De Bacquer D De Vriese AS. Vitamin K antagonists for stroke prevention in hemodialysis patients with atrial fibrillation: a systematic review and Meta-analysis. Am Heart J. (2017) 184:37–46. doi: 10.1016/j.ahj.2016.09.016 27892885

[26] Connolly SJ Ezekowitz MD Yusuf S Eikelboom J Oldgren J Parekh A . Dabigatran versus warfarin in patients with atrial fibrillation. N Engl J Med. (2009) 361:1139–51. doi: 10.1056/NEJMoa0905561 19717844

[27] Patel MR Mahaffey KW Garg J Pan G Singer DE Hacke W . Rivaroxaban versus warfarin in nonvalvular atrial fibrillation. N Engl J Med. (2011) 365:883–91. doi: 10.1056/NEJMoa1009638 21830957

[28] Granger CB Alexander JH McMurray JJ Lopes RD Hylek EM Hanna M . Apixaban versus warfarin in patients with atrial fibrillation. N Engl J Med. (2011) 365:981–92. doi: 10.1056/NEJMoa1107039 21870978

[29] Chen C Cao Y Zheng Y Dong Y Ma J Zhu W . Effect of rivaroxaban or apixaban in atrial fibrillation patients with stage 4-5 chronic kidney disease or on dialysis. Cardiovasc Drugs Ther. (2021) 35:273–81. doi: 10.1007/s10557-021-07144-8 33538928

[30] Cho MS Yun JE Park JJ Kim YJ Lee J Kim H . Outcomes after use of standard- and low-dose non-vitamin K oral anticoagulants in Asian patients with atrial fibrillation. Stroke. (2018) 50:110–118. doi: 10.1161/strokeaha.118.023093 30580716

[31] Nielsen PB Skjøth F Søgaard M Kjældgaard JN Lip GY Larsen TB. Effectiveness and safety of reduced dose non-vitamin K antagonist oral anticoagulants and warfarin in patients with atrial fibrillation: propensity weighted Nationwide cohort study. BMJ. (2017) 356:j510. doi: 10.1136/bmj.j510 28188243 PMC5421446

[32] Larsen TB Skjøth F Nielsen PB Kjældgaard JN Lip GY. Comparative effectiveness and safety of non-vitamin K antagonist oral anticoagulants and warfarin in patients with atrial fibrillation: propensity weighted Nationwide cohort study. BMJ. (2016) 353:i3189. doi: 10.1136/bmj.i3189 27312796 PMC4910696

[33] Schulman S Kearon C. Definition of major bleeding in clinical investigations of Antihemostatic medicinal products in non-surgical patients. J Thrombosis Hemostasis. (2005) 3:692–4. doi: 10.1111/j.1538-7836.2005.01204.x 15842354

[34] Chen HY Ou SH Huang CW Lee PT Chou KJ Lin PC . Efficacy and safety of direct oral anticoagulants vs warfarin in patients with chronic kidney disease and dialysis patients: a systematic review and meta-analysis. Clin Drug Investig. (2021) 41:341–51. doi: 10.1007/s40261-021-01016-7 33709339

[35] Siontis KC Zhang X Eckard A Bhave N Schaubel DE He K . Outcomes associated with Apixaban use in patients with end-stage kidney disease and atrial fibrillation in the United States. Circulation. (2018) 138:1519–29. doi: 10.1161/circulationaha.118.035418 29954737 PMC6202193

[36] Makani A Saba S Jain SK Bhonsale A Sharbaugh MS Thoma F . Safety and efficacy of direct oral anticoagulants versus warfarin in patients with chronic kidney disease and atrial fibrillation. Am J Cardiol. (2020) 125:210–4. doi: 10.1016/j.amjcard.2019.10.033 31780073

[37] Ruff CT Giugliano RP Braunwald E Hoffman EB Deenadayalu N Ezekowitz MD . Comparison of the efficacy and safety of new oral anticoagulants with warfarin in patients with atrial fibrillation: a meta-analysis of randomised trials. Lancet. (2014) 383:955–62. doi: 10.1016/s0140-6736(13)62343-0 24315724

[38] Miller CS Grandi SM Shimony A Filion KB Eisenberg MJ. Meta-analysis of efficacy and safety of new oral anticoagulants (dabigatran, rivaroxaban, Apixaban) versus warfarin in patients with atrial fibrillation. Am J Cardiol. (2012) 110:453–60. doi: 10.1016/j.amjcard.2012.03.049 22537354

[39] Mitchell SA Simon TA Raza S Jakouloff D Orme ME Lockhart I . The efficacy and safety of oral anticoagulants in warfarin-suitable patients with nonvalvular atrial fibrillation: systematic review and meta-analysis. Clin Appl Thromb Hemost. (2013) 19:619–31. doi: 10.1177/1076029613486539 23698729

[40] Bruins Slot KM Berge E. Factor Xa inhibitors versus vitamin K antagonists for preventing cerebral or systemic embolism in patients with atrial fibrillation. Cochrane Database Syst Rev. (2013) 8:Cd008980. doi: 10.1002/14651858.CD008980.pub2 23925867

[41] Aursulesei V Costache II. Anticoagulation in chronic kidney disease: from guidelines to clinical practice. Clin Cardiol. (2019) 42:774–82. doi: 10.1002/clc.23196 31102275 PMC6671778

[42] Kimachi M Furukawa TA Kimachi K Goto Y Fukuma S Fukuhara S. Direct oral anticoagulants versus warfarin for preventing stroke and systemic embolic events among atrial fibrillation patients with chronic kidney disease. Cochrane Database Syst Rev. (2017) 11:Cd011373. doi: 10.1002/14651858.CD011373.pub2 29105079 PMC6485997

[43] Feldberg J Patel P Farrell A Sivarajahkumar S Cameron K Ma J . A systematic review of direct oral anticoagulant use in chronic kidney disease and dialysis patients with atrial fibrillation. Nephrol Dialysis Trans. (2019) 34:265–77. doi: 10.1093/ndt/gfy031 29509922

[44] Ha JT Neuen BL Cheng LP Jun M Toyama T Gallagher MP . Benefits and harms of Oral anticoagulant therapy in chronic kidney disease: a systematic review and meta-analysis. Ann Intern Med. (2019) 171:181–9. doi: 10.7326/m19-0087 31307056

[45] Giugliano RP Ruff CT Braunwald E Murphy SA Wiviott SD Halperin JL . Edoxaban versus warfarin in patients with atrial fibrillation. N Engl J Med. (2013) 369:2093–104. doi: 10.1056/NEJMoa1310907 24251359

[46] Quinn LM Richardson R Cameron KJ Battistella M. Evaluating time in therapeutic range for hemodialysis patients taking warfarin. Clin Nephrol. (2015) 83:80–5. doi: 10.5414/cn108400 25500296

[47] De Vriese AS Caluwé R Van Der Meersch H De Boeck K De Bacquer D. Safety and efficacy of vitamin K antagonists versus rivaroxaban in hemodialysis patients with atrial fibrillation: a multicenter randomized controlled trial. J Am Soc Nephrol. (2021) 32:1474–83. doi: 10.1681/asn.2020111566 33753537 PMC8259651

