# Supplementary material for: Direct oral anti-Xa anticoagulants versus warfarin in newly diagnosed atrial fibrillation and CKD: the Korean National Health Insurance Data
Source: Front Med (Lausanne). 2023 Sep 15;10:1212816. doi: 10.3389/fmed.2023.1212816 (PMC10541953; doi:10.3389/fmed.2023.1212816)
Supplement: Supplementary file 1 [file Table_1.DOCX]

SUPPLEMENTAL MATERIAL

**Direct Oral Anti-Xa Anticoagulants** **versus Warfarin in newly diagnosed Atrial Fibrillation and CKD: the Korean National Health Insurance Data**

Youn Kyung Kee, M.D.^1^, Hee Jung Jeon, M.D., Ph.D. ^1^, Jieun Oh, M.D., Ph.D. ^1^, Tae-Hyn Yoo, M.D., Ph.D.^2^ ,Dongwoo Kang^3^, Jungkuk lee^3^, Dong Ho Shin, M.D., Ph.D. ^1,*^

**Author affiliations**

^1^Department of Internal Medicine, College of Medicine, Hallym University, Kangdong Sacred Heart Hospital, 150, Seongan-ro, Gangdong-gu, Seoul 05355 Korea

^2^ Department of Internal Medicine, College of Medicine, Yonsei University

^2^ Data Science Team, Hanmi Pharm. Co., Ltd

**Corresponding Author**

Dong Ho Shin, M.D., Ph.D.

Department of Internal Medicine, College of Medicine, Hallym University, Kangdong Sacred Heart Hospital, 150, Seongan-ro, Gangdong-gu, Seoul 05355 Korea

Tel; 82-2-2224-2900

Fax; 82-2-478-2825

E-mail: isaac9713@gmail.com

| Supplement Table 1. Diseases according to ICD-10 codes to extract study population | |
| --- | --- |
| Disease | ICD-codes |
| CKD | N18, N18.1, N18.3, N18.4, N18.5, N18.9 |
| Atrial fibrillation | I480, I481, I482, 489 |
| Mitral stenosis | I05.0, I05.2 |
| ^*^Mechanical valve | Z95.2-Z95.4 |
| ^*^Deep vein thrombosis | I80.2 |
| ^*^Pulmonary embolism | I26 |
| ^*^Any cancers | C00 - C97 |
| ^**,*^Intracranial hemorrhage | I60, I61, I62 |
| ^**,*^Gastrointestinal bleeding | I85.0, K22.1, K22.8, K25.0, K25.2, K25.4, K25.6, K26.0, K26.2, K26.4, K26.6, K27.0, K27.2, K27.4, K27.6, K28.0, K28.2, K28.4, K28.6 ,K29.0, K31.8, K55.2, K57.0, K57.1, K57.2, K57.3 K57.4 K57.5, K57.8, K57.9, K62.5, K66.1, K92.0, K92.1, K92.2 |
| ^**,*^Extracranial or unclassified major bleeding | H052, H356, H431, J942, M250, D02, R04 |
| ^**,*^Ischemic stroke | I63, I64 |
| ^*^Used in exclusion criteria. | |
| ^**^Used for outcomes measurement.  CKD, chronic kidney disease | |

| Supplement Table 2. Comorbidities according to ICD-10 codes and procedure codes | |  |
| --- | --- | --- |
| Disease | ICD-codes | ^*^Procedure codes |
| Pre-dialytic CKD | N18, N18.1, N18.3, N18.4, N18.9 |  |
| ESRD | N18.5 | O7001, O7002, O7003, O7004, O7005, O7011, O7012, O7013, O7014, O7015, O7016, O7017, O7018, O7020, O7021, O7031, O7032, O7033, O7034, O7035, O7061, O7062, O7074, O7075, O7076, O7077, O7080, O7081 |
| Diabetes mellitus with chromic complication | E10.0, E10.6, E10.8, E11.0, E11.6, E11.8, E12.0, E12.1, E12.6, E12.8, E12.9, E13.0, E13.6, E13.8, E14.0, E14.6, E14.8, E10.1, E10.9, E11.1, E11.9, E13.1, E13.9, 1E14.1, E14.9 |  |
| Diabetes mellitus without chromic complication | E10.5, E10.7, E11.5, E11.7, E12.2, E12.3, E12.4, E12.5, E12.7, E13.5, E13.7, E14.5, E14.7, E10.2, E10.3, E10.4, E11.2, E11.3, E11.4, E13.2, E13.3, E13.4, E14.2, E14.3, E14.4 |  |
| Congestive heart failure | I09.9, I11.0, I13.0, I13.2, I25.5, I42.0, I42.5, I42.6, I42.7, I42.8, I42.9, I43, P29.0, I50 |  |
| Myocardial infarction | I21, I22, I25.2 |  |
| Peripheral vascular disease | I70, I73.1, I73.8, I77.1, I79.0, I79.2, K55.1, K55.8, K55.9, I71, I73.9, Z95.8, Z95.9 |  |
| Mild liver disease | B18, K70.0, K70.1, K70.1, K70.2, K70.9, K71.3, K71.4, K71.5, K74.0, K74.1, K74.2, K76.0, K76.2, K76.3, K76.4, K76.8, K76.9, Z94.4, K70.3, K70.7, K73, K74.4, K74.5 |  |
| Moderate or severe liver disease | I85.0, I85.9, I86.4, I98.2, K70.4, K71.1, K76.5, K72.1, K72.9, K76.6, K76.7 |  |
| Peptic ulcer disease | K25.0, K25.1, K25.2, K25.3, K25.9, K26.0, K26.1, K26.2, K26.3, K26.9, K27.0, K27.1, K27.2, K27.9, K28.0, K28.2, K28.1, K28.2, K28.3, K28.9, K25.4, K25.5, K25.6, K25.7, K26.4, K26.5, K26.6, K26.7, K27.4, K27.5, K27.6, K27.7, K28.4, K28.5, K28.6, K28.7 |  |
| Hemiplegia or paraplegia | G04.1, G11.4, G80.1, G80.2, G82.3, G82.4, G82.5 G83.0, G83.1, G83.2, G83.3, G83.4, G83.9, G81, G82.0, G82.1, G82.2 |  |
| Dementia | F03, F05.1, G30, G31.1, F00, F01, F02 |  |
| AIDS | B24, B20, B21, B22 |  |

^*^Procedure codes for dialysis

CKD, chronic kidney disease; ESRD, end-stage renal disease; AIDS, acquired immunodeficiency syndrome

Supplement Table 3. Prescribed drug doses according to types of non-vitamin K antagonist oral anticoagulants

| Apixaban 2.5mg twice daily, n (%) | 60 (6.5) |
| --- | --- |
| Apixaban 5mg twice daily, n (%) | 247 (27.0) |
| Rivaroxaban 15mg daily, n (%) | 183 (20.0) |
| Rivaroxaban 20mg daily, n (%) | 178 (19.5) |
| Edoxaban 30mg daily, n (%) | 174 (19.0) |
| Edoxaban 60mg daily, n (%) | 73 (8.0) |

| Supplement Table 4. Baseline characteristics of subjects before and after propensity score matching | | | | | | |  |
| --- | --- | --- | --- | --- | --- | --- | --- |
|  | Before propensity score matching | | | After propensity score matching | | | |
|  | Warfarin user  (n =970) | DOAC user  (n =915) | P-value | Warfarin user  (n =120) | DOAC user  (n =120) | P-value | |
| Age, y | 68.4 ± 12.0 | 73.7 ± 10.1 | <0.001 | 72.5 ± 11.0 | 72.5 ± 10.7 | 0.82 | |
| Male, n (%) | 573 (59.1) | 487 (53.2) | <0.001 | 71 (59.2) | 63 (52.5) | 0.61 | |
| Comorbid disease, n (%) |  |  |  |  |  |  | |
| Pre-dialysis CKD | 655 (67.5) | 855 (93.4) | <0.001 | 80 (66.7) | 85 (70.8) | 0.45 | |
| ESRD | 315 (32.5) | 60 (6.6) | <0.001 | 40 (33.3) | 35 (29.2) | 0.56 | |
| Diabetes mellitus | 675 (69.6) | 665 (72.7) | 0.14 | 84 (70.0) | 87 (72.5) | 0.73 | |
| Congestive heart failure | 551 (56.8) | 522 (57.0) | 0.93 | 68 (56.7) | 69 (57.5) | 0.88 | |
| Myocardial infarction | 111 (11.4) | 111 (12.1) | 0.67 | 14 (11.7) | 13 (10.8) | 0.89 | |
| Peripheral vascular disease | 361 (37.2) | 412 (45.0) | 0.001 | 44 (36.7) | 48 (40.0) | 0.52 | |
| Charlson's comorbidity index | 4.08 ± 2.02 | 4.69 ± 2.04 | <0.001 | 4.40 ± 2.00 | 4.50 ± 2.05 | 0.69 | |
| CHA_2_DS_2_-VASc | 4.64 ± 1.63 | 5.17 ± 1.68 | <0.001 | 4.85 ± 1.60 | 5.00 ± 1.65 | 0.75 | |
| mHAS-BLED^*^ | 2.72 ± 0.90 | 3.07 ± 0.79 | <0.001 | 2.85 ± 0.85 | 2.90 ± 0.80 | 0.74 | |
| Medication, n (%) |  |  |  |  |  |  | |
| Aspirin | 449 (46.3) | 453 (49.5) | 0.17 | 55 (45.8) | 59 (49.2) | 0.72 | |
| Other antiplatelets | 178 (18.4) | 189 (20.7) | 0.22 | 21 (17.5) | 24 (20.0) | 0.60 | |
| BB | 521 (53.7) | 416 (45.5) | <0.001 | 64 (53.3) | 54 (45.0) | 0.25 | |
| CCB | 593 (61.1) | 570 (62.3) | 0.64 | 73 (60.8) | 74 (61.7) | 0.91 | |
| RASB | 687 (70.8) | 655 (71.6) | 0.72 | 83 (69.2) | 85 (70.8) | 0.85 | |
| Diuretics | 536 (55.3) | 520 (56.8) | 0.52 | 67 (55.8) | 68 (56.7) | 0.93 | |
| Statin | 465 (47.9) | 503 (55.0) | 0.002 | 56 (46.7) | 65 (54.2) | 0.26 | |
| DOACs, Direct Oral Anti-Xa Anticoagulants; RASB, renin-angiotensin-aldosterone system blocker; CCB, calcium channel blocker; BB, beta blocker  ^*^Modified HAS-BLED | | | | | | |  |

Supplement Table 5. Primary and secondary efficacy outcomes after propensity score matching

| Outcome | Group | Events | Person-year (per 1,000) | HR (95% CI) | P-value |
| --- | --- | --- | --- | --- | --- |
|  | (n) | (n) |  |  |  |
| Ischemic stroke | Warfarin user |  |  |  | - |
|  | 120 | 19 | 1.94 | ref |  |
|  | DOAC user |  |  |  |  |
|  | 120 | 18 | 1.88 | 0.94 (0.50 – 1.75) | 0.82 |
| All-cause death | Warfarin user |  |  |  | - |
|  | 120 | 30 | 2.05 | ref |  |
|  | DOAC user |  |  |  |  |
|  | 120 | 16 | 1.75 | 0.53 (0.30 – 0.95) | 0.03 |

HR, hazard ratio; DOACs, Direct Oral Anti-Xa Anticoagulants; CI, confidence interval

Supplement Table 6. Primary safety outcomes after propensity score matching

| Outcome | Group | Events | Person-year(per 1,000) | HR (95% CI) | P-value |
| --- | --- | --- | --- | --- | --- |
|  | (n) | (n) |  |  |  |
| Intracranial hemorrhage | Warfarin user |  |  |  | - |
|  | 120 | 15 | 2.50 | ref |  |
|  | DOAC user |  |  |  |  |
|  | 120 | 7 | 1.75 | 0.40 (0.16 – 0.99) | 0.05 |
| Gastrointestinal bleeding | Warfarin user |  |  |  | - |
|  | 120 | 45 | 2.25 | ref |  |
|  | DOAC user |  |  |  |  |
|  | 120 | 20 | 1.25 | 0.50 (0.29 – 0.86) | 0.001 |
| Extracranial or unclassified Major bleeding | Warfarin user |  |  |  | - |
|  | 120 | 38 | 2.30 | ref |  |
|  | DOAC user |  |  |  |  |
|  | 120 | 18 | 1.50 | 0.53 (0.30 – 0.93) | 0.03 |

HR, hazard ratio; DOACs, Direct Oral Anti-Xa Anticoagulants; CI, confidence interval
